# Social Determinants and the Classification of Disease: Descriptive Epidemiology of Selected Socially Mediated Disease Constellations

**DOI:** 10.1371/journal.pone.0110271

**Published:** 2014-11-05

**Authors:** Robert S. Levine, Barbara A. Kilbourne, George S. Rust, Michael A. Langston, Baqar A. Husaini, Lisaann S. Gittner, Maureen Sanderson, Charles H. Hennekens

**Affiliations:** 1 Department of Family and Community Medicine, Meharry Medical College, Nashville, TN, United States of America; 2 Department of Sociology, Tennessee State University, Nashville, TN, United States of America; 3 Department of Community and Preventive Medicine, Morehouse School of Medicine, Atlanta, GA, United States of America; 4 Department of Electrical Engineering and Information Technology, University of Tennessee, Knoxville, TN, United States of America; 5 Center for Prevention Research, Tennessee State University, Nashville, TN, United States of America; 6 Department of Political Science, Texas Tech University, Lubbock, TX, United States of America; 7 Charles E. Schmidt College of Medicine, Department of Epidemiology, Florida Atlantic University, Boca Raton, FL, United States of America; University of São Paulo School of Medicine, Brazil

## Abstract

**Background:**

Most major diseases have important social determinants. In this context, classification of disease based on etiologic or anatomic criteria may be neither mutually exclusive nor optimal.

**Methods and Findings:**

Units of analysis comprised large metropolitan central and fringe metropolitan counties with reliable mortality rates – (n = 416). Participants included infants and adults ages 25 to 64 years with selected causes of death (1999 to 2006). Exposures included that residential segregation and race-specific social deprivation variables. Main outcome measures were obtained via principal components analyses with an orthogonal rotation to identify a common factor. To discern whether the common factor was socially mediated, negative binomial multiple regression models were developed for which the dependent variable was the common factor. Results showed that infant deaths, mortality from assault, and malignant neoplasm of the trachea, bronchus and lung formed a common factor for race-gender groups (black/white and men/women). Regression analyses showed statistically significant, positive associations between low socio-economic status for all race-gender groups and this common factor.

**Conclusions:**

Between 1999 and 2006, deaths classified as “assault” and “lung cancer”, as well as “infant mortality” formed a socially mediated factor detectable in population but not individual data. Despite limitations related to death certificate data, the results contribute important information to the formulation of several hypotheses: (a) disease classifications based on anatomic or etiologic criteria fail to account for social determinants; (b) social forces produce demographically and possibly geographically distinct population-based disease constellations; and (c) the individual components of population-based disease constellations (e.g., lung cancer) are phenotypically comparable from one population to another but genotypically different, in part, because of socially mediated epigenetic variations. Additional research may produce new taxonomies that unify social determinants with anatomic and/or etiologic determinants. This may lead to improved medical management of individuals and populations.

## Introduction

For many years, social determinants have been known to affect morbidity and mortality from major diseases. In 1840, for example, Villerme [1] observed that people of higher social status, represented by occupational positions in management and merchandizing, could expect to live 28.2 years, while those holding factory jobs could expect to live only 17.6 years. [2] Today, social determinants continue to have a major impact on major causes of death [3] despite the transition from dominance of infectious diseases to dominance of chronic diseases. [4].

Nonetheless, the most commonly used method of disease classification primarily reflects individual anatomic and/or etiologic categories. Specifically, the International Classification of Diseases (ICD) [5], [6] is the standard tool for uses that range from individual clinical diagnoses to the health of the general public. [7] According to ICD criteria, disease definitions are mutually exclusive. [8] In this regard it is important to note that the basic structure of the ICD was established in 1898 [8] and was strongly influenced by the germ theory of disease as promoted by leading scientists of the age like Louis Pasteur and Robert Koch. At that time, it was believed that micro-organisms such as the tubercle bacillus were the fundamental causes of diseases such as tuberculosis. At present, however, it is generally recognized that factors such as micro-organisms are necessary but not sufficient causes of disease. [9] Social determinants acting long before the disease manifests clinically are fundamental causes, [4] and infectious diseases such as tuberculosis and cholera share a common social foundation with cardiovascular disease and cancer. [4] In this context, when viewed through a social lens, definitions of disease based on etiologic or anatomic criteria may be neither mutually exclusive nor optimal.

In the present report, we explore these issues using infant mortality which reflects general population health because of its strong relationship with such social determinants as economic development, general living conditions, social well-being and environmental quality. [10].

## Methods

### Data Sources

County-level estimates of infant mortality and other causes of death were obtained from the Compressed Mortality File for 1999 to 2006 as presented on the US Centers for Disease Control and Prevention’s publically available Wide-ranging Online Data for Epidemiologic Research (WONDER) internet web site. [11] Demographic data was obtained from two sources, including the Year 2000 US Decennial Census, as compiled with Geolytics software, [12] and indices of county-level residential segregation from the University of Arizona Geo-coding project. [13], [14] To identify counties with sufficient numbers of deaths from all causes to meet standards of reliability, which we defined as at least 20 deaths among infants and persons 25 to 64 years of age over the 8-year period. Units of analysis were restricted to counties classified by the US Census as large metropolitan central counties and large metropolitan fringe counties (overall n = 416).

### Analyses

After preliminary exploration to identify causes of death for which there were positive, statistically significant zero-order correlations with county levels of infant mortality across all race-gender groups (not shown), we performed principal components analyses with an orthogonal rotation per SAS PROC Factor. [15] A common factor was identified from among the following disease constellations: death and chronic respiratory disease; transport accidents; other external accidents; and cerebrovascular disease (stroke). The identified common factor for all race-gender groups includes: (1) infant mortality; (2) assault; (3) malignant neoplasm of the trachea, bronchus and lung (lung cancer-age-adjusted (25–64 years) race sex adjusted rates). This will subsequently be referred to as the common factor. To discern whether the common factor was socially mediated by socio-economic deprivation, multiple regression models were developed for which the dependent variable was the common factor. The common factor was developed using principal components analysis of age adjusted, race sex specific rates each individual disease component of the r constellations. Since the distributional characteristics common factor violated assumptions for Ordinary Least Squares, regression and comparison of Poisson and negative binomial models indicated under-dispersion, negative binomial regression (SAS, v9.23, PROC GENMOD) [16] was used.

The independent variables included composite measures of race-specific deprivation. The deprivation variables were adapted from Krieger et al’s [17] socio-economic positioning index (SEP). We included only those SEP items that predicted deprivation rather than privilege using county-level estimates from the US Census. In addition, we added a measure of racial residential segregation (Black Isolation Index and White Isolation Index for residential segregation in 2000. [13], [14] We used PCA (Principal Components Analysis, SAS V9.23, Proc Factor) [15] to analyze race-specific items from the 2000 Census; orthogonal rotation was used to construct the race-specific deprivation composite measures. Only items with loadings greater than 0.75 on factors for both races were selected for inclusion. This yielded two composite measures or indices, each including two variables, and a single measure of race-specific segregation. The first was the average percentage of households with incomes less than poverty and adults attaining less than high school education. The second included the percentage of households renting and households with no access to an automobile.

## Results

Table 1 shows initial zero-order race- gender group correlations between race-specific infant mortality and causes of death in non-Hispanic populations. Most causes of death were significantly correlated with infant mortality across race/sex groups. Correlations were stronger in whites for both genders and were the strongest for white men. Only three correlations failed to achieve statistical significance, Infant mortality and transport accidents among blacks was not significant among either black men or women, and among black women, the collective category, other external causes of death, was not correlated with infant mortality.

**Table 1:** Race-gender-specific correlations between age-adjusted (25 to 64 years) mortality from selected causes of death and race-specific infant mortality (zero order) for non-Hispanics in 416 US Central and Fringe Metropolitan Counties, 1999–2010.

*Underlying Cause of Death*	*White Male*	*White Female*	*Black Male*	*Black Female*
Assault	0.62***	0.52***	0.41***	0.28*
Malignant Neoplasm of the Trachea, Bronchus and Lung	0.64***	0.37***	0.40***	0.30***
Chronic Lower Respiratory Disease	0.59***	0.41***	0.30***	0.17*
Transport Accident	0.50***	0.46***	0.03	−0.02
Other External Accident	0.46***	0.45***	0.24***	0.10
Cerebrovascular Accident (Stroke)	0.42***	0.32***	0.25***	0.30***

*** = p<0.001; * = p<0.05.

Table 2 shows the results of Principal Components Analyses. All factors share only three elements that were significantly correlated across all race-gender groups to form a Common Factor: infant mortality, assault, and malignant neoplasm of the trachea, bronchus and lung (lung cancer). Additionally, when taken separately, each race-gender group yielded significantly different factor structures.

**Table 2:** Factor loading and alpha-reliability from principal components analysis.

Race-gender Group				Cause of Death			
	Infant Mor-tality	Malignant Neoplasm of the Trachea,Bronchus, and Lung	Assault	Chronic LowerRespira-tory Disease	Other ExternalAccidents	TransportAccidents	Cerebro-VascularAccidents
White Males (Alpha 0.88)	0.78	0.87	0.79	0.82	0.63	0.78	
Black Males (Alpha 0.78)	0.83	0.68	0.83		0.77		
White Females (Alpha 0.85)	0.60	0.71	0.73	0.62	0.69		0.76
Black Females (Alpha 0.67)	0.72	0.78	0.71				0.65

Table 3 shows the results of negative binomial regression for which the dependent variable was the average of mortality for causes of death included in the common factor. This showed deleterious effects for poverty and low educational attainment in all race-gender groups. These effects were significantly greater among whites. The effects for poverty and low educational attainment on mortality associated with the common factor were 95 fold for white men and 67 fold for white women. Among blacks, deaths associated with the common factor were 4.1 fold for men and 5.9 fold for women. The rental/crowding index appeared slightly but significantly protective for all race sex groups, reducing associated mortality associated by one to two percent. Residential segregation decreased risks in whites by roughly 38% and increased risks in black men. Racial segregation did not significantly affect mortality risk for black women. Examination of the standard errors associated with the coefficients suggest this lack of significance is likely due to relatively higher degree of correlation between segregation and the poverty/low educational attainment for this race/sex group.

**Table 3:** Estimates of the effects of social determinants on common factor mortality.

RACE-GENDER GROUP	BLACK MALES	BLACK FEMALES	WHITE MALES	WHITE FEMALES
Race-Specific Poverty and Low Education Index	4.109	5.971	95.231	66.993
	(2.533–6.667)	(2.735–13.037)	(50.571–179.331)	(29.848–150.367)
	p<0.0001	P<0.0001	P<0.0001	P<0.0001
Race-Specific % Renters and Crowding Index	0.990	0.981	0.988	0.991
	(0.986–0.996)	(0.975–0.988)	(0.982–0.994)	(0.984–0.997)
	p<0.0001	p<0.0001	p<0.0001	p<0.0001
Race-Specific Isolation Index	1.381	1.054	0.62181	0.624
	(1.175–1.623)	(0.841–1.321)	(0.483–0.801)	(0.474–0.819)
	p<0.0001	p = 0.6502	p = 0.0002	p = 0.0007
n	137	80	198	175

Exponentiated coefficients and 95% confidence intervals from negative binomial regression.

**Figure 1:**
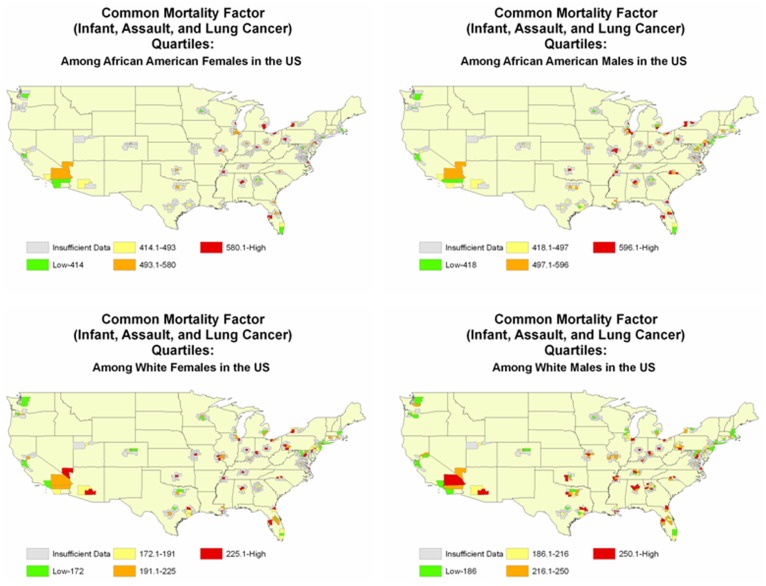
Race-gender specific geographic distribution of the Common Factor. USA. 1999–2006.

Finally, as shown in Figure 1, cities in the lowest quartiles of common factor mortality rates for all race-gender groups included Oakland, CA; Fort Lauderdale, FL; Manhattan, NY; and Seattle, WA. Cities in the highest quartile for all race-gender groups included Birmingham, AL; Cincinnati, OH; Norfolk, VA; and Philadelphia, PA.

Data used for the analyses in this paper is available as supporting information (Text S1. Social Determinants Data).

## Comment

In these data, “lung cancer” and “assault” were not mutually exclusive causes of death between 1999 and 2006. Although no one person ages 25 to 64 years in these 416 counties could simultaneously experience lung cancer, assault, and infant mortality, population-based analysis did reveal a socially mediated link between these conditions. Thus, in this context, consideration of “assault” and “lung cancer” as mutually exclusive terms is erroneous because all three diseases have a common causal web of a larger socially-mediated structure. There is a core disease constellation (infant mortality-assault-lung cancer) present for blacks, whites, men and women, but only apparent at the population level. Moreover, this constellation occurred throughout United States central metropolitan and fringe metropolitan areas at varying rates, meaning that it may exert different influences on socially mediated disease from one place to another.

The link between infant mortality, assault, and lung cancer is consistent with observations that mortality from both assault (men) and lung cancer (both genders) are predictable based on low childhood socio-economic status which may, in turn, increase the likelihood of acquiring risky behaviors. [18] Risky behaviors plausibly include smoking and a propensity for assault with firearms. Further, the association between maternal smoking and infant mortality has been consistently observed, both within the US and abroad.[19]–[23].

Disease classifications based solely on anatomic/etiologic criteria that fail to account for social determinants have several major limitations. First, as noted above, the data show that disease classifications based solely on anatomic/etiologic criteria are not, in fact, mutually exclusive terms. Additionally, as shown in Table 2, the population-level configuration of causes of death which are grouped with lung cancer may vary according to socio-demographic characteristics rather than characteristics determined by anatomy or etiology. Nonetheless, lung cancer and other disease classifications based on anatomy and etiology have indisputable clinical value. A possible way to reconcile this apparent contradiction is that the socio-demographically stable clinical/individual/anatomic-etiologic classification represents the phenotype of lung cancer, while the socially mediated and variable population-level groupings reflect variations based on non-genomic heritability. [24] Non-genomic heritability is, in part, an adaptive epigenetic mechanism that involves regulation of genes due to environmental factors encompassing social determinants of health. In the case of lung cancer, there is evidence that socio-economic status is closely related to DNA methylation profiles, [25] and that epigenetic alterations like promoter DNA methylation leading to gene silencing are common.[26.27] It would be of interest to test the hypothesis that place- or person-based variations in disease constellations such as those appearing in Table 2 are associated with epigenetic variations affecting the occurrence of lung cancer.

It is plausible that while lung cancer remains phenotypically identical from one social context to another, the race-gender specific epigenetic foundations of lung cancer reflect differences in genotype based on epigenetic programming. Thus the lung cancer which occurs in black men as part of the “infant mortality-assault-lung cancer-external accidents” grouping may differ from that of phenotypic lung cancer which occurs from a different disease constellation combination, such as the one we have observed in white women. Programming of the epigenome during pregnancy and early life derives from both parents and is a critical determinant of later life disease outcomes, standing at the interface between the environment and genetics. [28] Based on the race-gender specific factors, comparable arguments could also be developed for infant mortality and assault based on epigenetic variation. [29] This may have clinical implications in that an individual’s capacity to respond to treatment might relate, in part, to the epigenetic programming underlying a particular phenotypic expression.

We believe that our findings are compatible with the hypothesis that those with greater access to resources are better able to avoid poor health outcomes. [30] We hypothesize, however, that both social and biological forms of non-genomic heritability are involved. The former may reflect transgenerational repetition of adaptive behaviors, [24] while the latter may reflect epigenetic modifications. [26] Transgenerational repetition of behavior by itself offers a primarily environmental explanation for the fundamental nature of social determinants. In contrast, Rothman et al. note that, “Every case of every disease has some environmental and some genetic component causes, and therefore every case can be attributed both to genes and to environment. No paradox exists as long as it is understood that the fractions of disease attributable to genes and to environment overlap with one another.” [31] The present data suggests the possibility that non-genomic epigenetic heritability may also play a role in the fundamental actions of social determinants on disease outcomes. While the present data do not include transgenerational information, this hypothesis is consistent with previous observations of biologically mediated, transgenerational transmission of socially mediated adverse health effects. [32], [33].

In summary, epigenetically derived phenotypes of lung cancer may vary according to race-gender social determinants, ultimately generating clinical benefits for both individuals and populations. From an individual perspective, phenotypic lung cancer may be clinically managed in a comparable manner wherever an individual resides. Improved medical knowledge of varying socially-mediated genotypes, however, might lead to even better management. From a public health and health policy perspective, population-based treatment/prevention programs for assault, lung cancer, and infant mortality as distinct entities might lead to different, and possibly less effective interventions than those based on disease constellations which vary around a core common factor (such as the one described here).

These descriptive data have several limitations. First, death-certificate data have well-known limitations. [34] For example although assault, lung cancer and infant mortality are universally recorded with reasonable accuracy, [35], [36] the same is not true for all causes of death. [36] Second, the data are based on the underlying cause of death, which is the condition which the attending physician identifies as the primary reason for an individual’s demise. Had the data included all conditions experienced by the individual at the time of death, different disease constellations might have been identified. Third, we did not search all possible zero-order correlates of infant mortality since the primary objective was to provide sufficient evidence to generate new hypotheses. More comprehensive searches might disclose additional disease constellations. Fourth, mortality statistics limit the data which can be included in the analyses, partly because even common causes of death may be relatively rare events. For example, by including assault in the analyses we had to limit our scope of study to the large metropolitan central and fringe metropolitan counties (because smaller counties did not regularly have ≥20 assault deaths among 25 to 64 year olds during the observation period). In contrast, searches using population-based morbidity data might be able to identify disease constellations across all types of communities in the urban-rural spectrum.

Despite these and other limitations, we believe the data contribute importantly relevant information to the formulation of a number of hypotheses: (a) disease classifications based on anatomic or etiologic criteria fail to account for social determinants; (b) social forces produce demographically and possibly geographically distinct population-based disease constellations; and (c) the individual components of population-based disease constellations (e.g., lung cancer) are phenotypically comparable from one population to another but genotypically different, in part, because of socially mediated epigenetic variations.

Further research is necessary which includes the possibility of new taxonomies to account for social as well as medical determinants of disease. The fundamental structure of such new taxonomies might be informed by classifications such as the system developed by Diderichsen et al. [37] which includes: 1) early determinants affecting social position and health (early childhood development, schooling, and segregation by the local community), 2) determinants of illness affected by social position (income and poverty, long-term unemployment, social marginalization, physical environment, work environment, health behavior, and early functional decline) and 3) determinants generating unequal consequences of illness (health services utilization and the exclusionary labor market).

Ultimately, new taxonomies might help to unify social and medical conceptualizations of disease and health. For all researchers, delineations of socially driven disease constellations might lead to a better understanding of the pathophysiologic pathways from poverty to illness. For public health planners and policy makers, conceptualizations of disease which unite social and traditional medical classifications might lead to interventions which are more successful at reducing or eliminating disparities than interventions based on a medical taxonomy alone.

## Supporting Information

Text S1 (ZIP)
